# Psychedelic-Induced Neural Plasticity: A Comprehensive Review and a Discussion of Clinical Implications

**DOI:** 10.3390/brainsci15020117

**Published:** 2025-01-25

**Authors:** Francesco Weiss, Anna Magnesa, Matteo Gambini, Riccardo Gurrieri, Eric Annuzzi, Camilla Elefante, Giulio Perugi, Donatella Marazziti

**Affiliations:** Department of Clinical and Experimental Medicine, Section of Psychiatry, University of Pisa, Via Roma 57, 56100 Pisa, Italy; francesco.weiss93@gmail.com (F.W.); anna.magnesa@gmail.com (A.M.); gambinimatteo1996@gmail.com (M.G.); riccardogurrieri4@gmail.com (R.G.); annuzzi.eric@gmail.com (E.A.); camifele@gmail.com (C.E.); giulio.perugi@med.unipi.it (G.P.)

**Keywords:** plasticity, 5-HT2A agonists, default-mode network, entropic brain, unlearning

## Abstract

**Background**: Psychedelics are increasingly recognized as a promising and innovative treatment strategy for several mental disorders. However, there is still a lively controversy in the medical community as regards the rationale of their employment, specifically their indications and potential dangers. **Methods**: A comprehensive literature search on “MEDLINE/PubMed” and “Web of Science” was performed from inception to 26 June 2024, cross-checking the obtained references. We included all studies, i.e., both clinical and preclinical, that supplied original data. **Results**: We initially obtained a total of 1083 entries, 813 from MEDLINE/PubMed and 270 from Web of Science. After duplicate elimination, 903 underwent systematic literature selection. Primary abstract screening yielded a total of 572 candidates for eligibility assessment and excluded 331 entries on formal grounds. Eligibility assessment led to the exclusion of 501 titles. Finally, a total of 70 articles were included in this review. **Discussion**: Preclinical evidence from genetic expression, histology and behavioral studies is soundly consistent with psychedelics possessing neuroplasticity-inducing properties. Despite methodological difficulties, clinical evidence seems to be inferentially in agreement with preclinical findings. However, it is still unclear whether the “neuroplastic boost” induced by classic psychedelics might be dissociable from the psychodysleptic effects, thereby reducing the psychopathological hazards implied by these compounds. Moreover, the fact that the so-called “relaxation of priors” should be unconditionally beneficial appears debatable, and further research should clarify the possible indications and contraindications of psychedelic psychoplastogens within a precision medicine perspective.

## 1. Introduction

The idea that compounds manifesting “antidepressant” properties might induce secondary molecular changes downstream of the immediate mechanism of reuptake inhibition dates back to 1970s, with Vetulani and Sulser’s seminal observations concerning the desensitization of β-adrenergic receptors after prolonged exposure to tricyclic antidepressants [1]. Although of little clinical consequence per se, these findings paved the way to a new mode of conceiving the mechanism of action of antidepressants [2], moving the focus from immediate neurochemical effects to mid-to-long term molecular adaptations that allegedly produced sustained modifications of neurons’ excitability and provided a rational explanation for the well-known thymoanaleptic latency of classic slow-acting antidepressants (SAADs). During the 90s, evidence was gathered relative to the desensitization of type 1 (5-HT1) and type 2 (5-HT2) serotonin receptors after weeks-long exposure to serotonergic agents [3,4].

In the years immediately following, preclinical inquiries commenced to suggest that all treatments effective against depression, namely ADs, sleep deprivation and electroconvulsive therapy, could increase the expression of a neurotrophin, the so-called brain-derived neurotrophic factor (BDNF) and its receptor (tropomyosin receptor kinase B, TrkB) in the rat hippocampus [5,6]. Neurotrophins (namely, nerve growth factor or NGF, BDNF, neurotrophins 3 and 4) are a class of growth factors that are crucial for neuronal survival, maturation and remodeling [7]. BDNF in particular seems to be crucial for those processes (i.e., synaptogenesis, dendritogenesis, spinogenesis) that allow for the unremitting adaptation of the brain to changing functional demands, briefly referred to as neuroplasticity [8]. In further support, some years later, an association between low BDNF plasma levels and major depression was being repeatedly reported [9,10,11,12].

Such data were particularly intriguing, since a number of magnetic resonance neuroimaging studies increasingly reported a reduction in hippocampal volumes in depressed patients [13,14,15,16]. Taken together, these accounts led to a reconceptualization of depression as a disorder of neuroplasticity: the so-called “network hypothesis” [17,18]. Consistently, monoaminergic potentiation and receptor adaptation started to be viewed as proximal precursory mechanisms of the real therapeutic action of ADs which was to increase neural growth and remodeling [19,20,21,22]. The demonstration that chronic administration the SSRI fluoxetine could restore plasticity in the visual cortex of amblyopic rats represented the first in vivo demonstration of a direct link between antidepressants and increased neuro-synaptogenesis [23], as supported thereafter in other studies [24,25]. Castrén interpreted the effect of antidepressants on brain remodeling as a reopening of the critical period of activity-dependent plasticity that physiologically occurs during early life and termed it “induced juvenile-like plasticity”, or “iPlasticity” for short [26,27,28].

Almost contemporarily, during the first decade of the 21st century, ketamine was shown to exert antidepressant properties in humans [29], being effective also in previously treatment-resistant patients [30]. Inasmuch ketamine acts mainly as an N-methyl-D-aspartate (NMDA) receptor antagonist and the clinical response occurs within hours of administration, its mechanism of action appears markedly different from that of SAADs and has readily enticed fervid speculations about its mode of action. Ketamine became the first “rapid-acting antidepressant” (RAAD, [31]) in the current sense of the term, and its effectiveness was soon attributed to its capacity to induce BDNF expression in various areas of the brain, thereby promoting synaptic growth and remodeling [32]. In the following years, other preclinical studies replicated the finding that BDNF is required for the rapid antidepressant action of ketamine and its enantiomers [33,34,35].

Ketamine aside, other promising RAADs, on which research has been concentrating in the last years, include scopolamine [36] and classic psychedelics, of which psilocybin boasts the largest body of evidence [37]. Heterogeneous as they might be, both scopolamine and psychedelics seem to require neuroplastic mechanisms (generally epitomized by BDNF synthesis and release) to exert their rapid antidepressant effects [38,39]. Not coincidentally, David E. Olson [40] recently coined the term “psychoplastogen” to refer to ketamine, scopolamine and psychedelics as a neuropharmacological class. However, “psycholastogenesis” and the subsequent “network rewiring” seem to be a final common pathway of both SAADs and RAADs (as well as electroconvulsive therapy), providing a putative neurobiological rationale for almost every antidepressant treatment [26,27].

However, some of the new antidepressants, namely psychedelics, still represent a controversial issue in the medical debate, with particular regard to their potential harms in the long term and the numerous methodological frailties found in the largest part of trials published until today [41,42,43]. We drafted the present work since we felt that a comprehensive review dealing specifically with the psychoplastogenic effects of classic psychedelics would contribute to dissolve part of the controversies, organizing and clarifying the evidence concerning how these substances share what seems to have become a definitory characteristic of all psychotropics with antidepressant effects.

## 2. Methods

The a priori goal of this review was to gather and analyze both clinical and preclinical literature pertaining to the psychoplastogenic properties of classic psychedelics, defined as 5-HT2A agonists and including mainly four compounds: N,N-dimethyltryptamine (N,N-DMT), mescaline, lysergic acid diethylamide (LSD) or lysergide and psilocin (and its more commonly known prodrug, psilocybin). Since we wanted to highlight the relationship between the specific mechanism of action of these substances and their neuroplastic properties, other compounds traditionally related to classic psychedelics but featuring different pharmacodynamics and phenomenality were discarded.

These latter include non-psychedelic hallucinogens such as entactogens like 3,4-methylenedioxy-methamphetamine (MDMA or midomafetamine), dissociatives like ketamine and phencyclidine, oneirogens like ibogaine and salvinorin A or deliriants like scopolamine and atropine [44]. On the other hand, modern synthetic compounds not comprised within the classic tetrad of psychedelics but sharing the same mechanism of action (such as 2,5-Dimethoxy-4-iodoamphetamine, or DOI) were taken into consideration.

First, a comprehensive literature search on “MEDLINE/PubMed” and “Web of Science” was performed from inception to 26 June 2024, cross-checking the obtained references. The systematic search was conducted using the following search string: “(psychedelic OR dmt OR dimethyltryptamine OR lsd OR lysergic acid diethylamide OR psilocybin OR psilocin OR mescaline) AND (plasticity OR neuroplasticity OR metaplasticity)”.

We included all studies, i.e., both clinical and preclinical, that supply original data. We concurrently posited the following formal exclusion criteria: non-English articles; review articles (including narrative and systematic reviews, pooled analyses and meta-analyses); books and book chapters; expert opinions or consensus articles; other documents not exhibiting original data (including editorials, consensus, expert opinion, guidelines, study protocols etc.).

To help us carry out the screening and organize the results, we made use of the online software Rayyan, copyright © 2022, https://new.rayyan.ai/ [45]. Both abstract screening and eligibility assessment were conducted by four authors independently (F.W., A.M., M.G. and R.G.), and conflicts were resolved with the aid of a fifth author (C.E.).

## 3. Results

**Figure 1:**
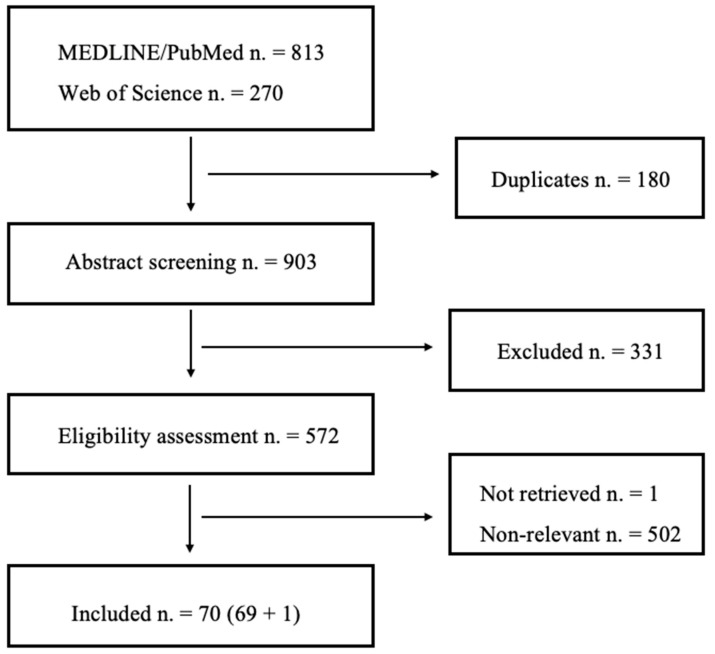
Flow diagram of literature screening.

With our research string, we initially obtained a total of 1083 entries, 813 from MEDLINE/PubMed and 270 from Web of Science. After duplicate elimination, 903 underwent systematic literature selection. Primary abstract screening yielded a total of 572 candidates for eligibility assessment and excluded 331 entries on formal grounds: non-English articles; review articles (including narrative and systematic reviews, pooled analyses and meta-analyses); books and book chapters; expert opinions or consensus articles; other documents not exhibiting original data (including editorials, consensus, expert opinion, guidelines, study protocols etc.). We were not able to retrieve one article [46]. Eligibility assessment led to the exclusion of 501 titles, among which were two articles concerning harmala alkaloids [47,48], and finally a total of 69 articles were initially included in this review. After snowball search, a further entry (n = 1) was included [49], leading to a total of 70 articles (the process is summarized in Figure 1).

The vast majority of the included papers involve preclinical models (n = 55), although a certain number were in vivo experiences in human subjects (n = 15). The oldest studies included date back to 1996–2000 and deal with how psychedelic substances influence long-term potentiation [50,51]. In order to clearly present the available information, we subdivided preclinical studies according to the compounds employed in the experimental design (psilocybin/psilocin, DMT-related compounds, LSD, mescaline and related compounds, synthetic 5-HT2A agonists such as DOI and 25C-NBOMe) and clinical studies based on methodology (neuroimaging studies, electrophysiological studies, biohumoral studies, behavioral studies and case reports).

### 3.1. Preclinical Evidence

Within the text, we opted to enumerate preclinical studies for each compound based on the chronological order of publication. The same evidence is summarized in Table 1, Table 2, Table 3, Table 4, Table 5 and Table 6 based on the design of studies and the techniques therein employed (upward arrows mean “increased” and downward arrows mean “decreased”).

#### 3.1.1. Psilocybin/Psilocin

A total of 20 preclinical papers dealing with the psychoplastogenic effects of psilocybin that were published from 2021 onwards were included. A post mortem autoradiography study displayed a statistically significant increase in a marker of synaptic density and plasticity (synaptic vesicle protein 2A, SV2A) and a decrease in 5-HT2A receptors both in the hippocampus and in the prefrontal cortex of pigs previously treated with 0.08 mg/kg intravenous psilocybin compared to placebo [52]. A study in mice treated with 1 mg/kg intraperitoneal psilocybin by using in vivo two-photon microscopy demonstrated that the active compound induced a persistent increase in the size and density of cortical dendritic spines lasting at least one month [53].

A quantitative PCR (qPCR) and Western blot (WB) approach in rats found that psilocybin induces rapid transcriptional modifications both in the prefrontal cortex and in the hippocampus, although the effect size was greater in the former. In particular, psilocybin was shown to upregulate plasticity-related genes (including Cebpb, c-Fos, Dups1, Fosb, Junb, Iκβ-α, Nr4a1, P11, Psd95 and Sgk1) in the same areas in a dose-dependent fashion [54]. Another work reported an ancillary result that psilocybin induces the transcription of plasticity-related genes, such as nptn, BDNF and Negr1, that were significant only in the prefrontal cortex [55].

A different study with murine models dealt with the influence of psilocin on sleep architecture through electroencephalography and local field potentials recordings [56], as the authors argued that the increase in oscillatory power around 4 Hz observed in psilocin-treated mice could be related to its psychoplastogenic properties. Indeed, these frequencies are known to be associated with either local sleep or prefrontal respiratory rhythm phenomena, which are in turn thought to be associated with neuroplastic processes [57,58]. Another work using rat models, albeit not strictly devoted to the topic, found as an ancillary result that psilocybin induces the transcription of plasticity-related genes, such as nptn, BDNF and Negr1 [59]. These effects were significant only in the prefrontal cortex and not in the hippocampus.

Again, psilocybin was shown to reduce the decrease of hippocampal dendritic complexity, spine density and BDNF expression in mice undergoing a fear conditioning (FC) test [60]. These histological and molecular effects paralleled a reduction in the increase in freezing time induced by FC at 24 h and on the 6th day and 7th day after psilocybin exposure. Another experiment with mice, focusing on IEG c-Fos, showed that psilocybin produced an acute increase of its expression in numerous brain regions, including the anterior cingulate cortex, the central and basolateral amygdala and the medial and lateral habenula [61]. Such an increase was comparable to that induced by ketamine and correlated with the endogenous levels of expression of NMDA receptor genes. Similarly, an immunofluorescence study in a murine model showed that psilocybin significantly increased c-Fos expression in subregions of the neocortex, caudoputamen tail and central amygdala, but a decreased expression in the hypothalamus, cortical amygdala, striatum and pallidum [62]. A study using a social reward-conditioned place preference (sCPP) model in mice showed that psilocybin was able to induce a critical period reopening, whose duration was similar to that of MDMA (about two weeks) but longer than ketamine (about 48 h) and shorter than LSD (about three weeks) and ibogaine (longer than four weeks) [63].

An immunohistochemical study demonstrated a dose-dependent increased c-Fos expression in neurons and oligodendrocytes located in the frontal cortex, nucleus accumbens, central and basolateral amygdala and locus coeruleus of rats previously administered psilocybin compared to vehicle [64]. A WB study in mice showed an increased expression of molecular biomarkers of synaptic plasticity (SV2A, GAP43, synaptophysin) 11 days after the administration of both synthetic psilocybin and mushroom extract in various brain areas including the prefrontal cortex, hippocampus and amygdala [65]. Another experiment using a qPCR managed to demonstrate a significantly increased expression of two IEGs (c-Fos and egr1) an hour after exposure to both synthetic psilocybin and mushroom extract in murine models [66]. A study using a murine conditioned fear model showed that the psilocin analogue 4-Hydroxy-N,N-diisopropyltryptamine (4-OH-DiPT) promoted fear extinction and spontaneous inhibitory post-synaptic currents (sIPSC) in basolateral amygdala [67].

A study in mice, making use of multiple techniques (including WB, immunofluorescence and dendrite analysis after Golgi staining), found that a single psilocybin administration reversed the detrimental effects of chronic glucocorticoid exposure on structural plasticity (in terms of density and number of dendritic spines or branches) and the expression plasticity-related proteins such as BDNF and TrkB [68]. Psilocybin was also demonstrated to exert “re-learning effects” in an experiment on rats using a drug-based affective bias protocol, not only reducing negative biases similarly to ketamine, but also uniquely increasing positive biases [69]. Interestingly, an experiment using a chronic despair model in 5-HT2A-deficient mice showed that psilocybin was still able to induce antidepressant effects, whereas DOI and lisuride did not [70]. Similarly, another study found that the 5-HT2A antagonist volinanserin abolished all of the antidepressant/anxiolytic behavioral effects of DOI and TCB-2 in rodents, whereas some effects of psilocin still persisted and were arguably independent of 5-HT2A-mediated signaling [71]. Finally, a preprint study claims to have identified a specific, regionally differentiated, drug-induced expression signature of c-Fos that allowed to discriminate psilocybin from 5-MeO-DMT, ketamine and MDMA with >95% accuracy [72].

**Table 1:** Gene expression studies: psilocybin/psilocin.

References	Methods	Results	Comments
Raval et al., 2021 [52] doi:10.3390/ijms22020835	Single dose of 0.08 mg/kg psilocybin in pigs In vitro autoradiography	Psilocybin increases SV2A expression in the hippocampus one and seven days post-injection.	Psilocybin significantly increases the expression of a marker of synaptic plasticity (synaptic vesicle protein 2A, SV2A) in the hippocampus.
Jefsen et al., 2021 [54] doi:10.1177/0269881120959614	Single dose of psilocybin (0.5–20 mg/kg) in rats Real-time qPCR	Prefrontal cortex: Cebpb↑, cFos↑, Dusp1↑, Fosb↑, Junb↑, Iκβ↑, Nr4a1↑, P11↑, Psd95↑, Sgk1↑, Clk1↓ Hippocampus: Arrdc2↑, Dusp1↑, Iκβ-α↑, Sgk1↑ Arc↓, Clk1↓, Egr2↓, Ptgs2↓	Psilocybin acutely and dose-dependently induces the expression of genes associated with neuroplasticity in the prefrontal cortex and the hippocampus.
Fadahunsi et al., 2022 [55] doi:10.1038/s41398-022-02103-9	Mice were randomized in four groups (intra-peritoneal injection of 0.3 mg/kg psilocybin, 1 mg/kg psilocybin, 3 mg/kg psilocybin, or isotonic saline) Real-time qPCR	Prefrontal cortex: Nptn↑, BDNF↑, Negr1↑	Psilocybin acutely and dose-dependently induces the expression of genes associated with neuroplasticity in the prefrontal cortex.
Liu et al., 2023 [59] doi:10.3389/fnins.2023.1168911	Single dose of 2.0 mg/kg psilocybin hydrochloride Immunofluorescence	EGR1↑	Psilocybin acutely increases the expression of EGR1, a gene involved in neuronal plasticity, across the brain.
Du et al., 2023 doi:10.1097/CM9.0000000000002647 [60]	Vehicle (sterile 0.9% saline) or psilocybin (0.1 mg/kg, 0.5 mg/kg, or 2.5 mg/kg) administered 30 min prior to Fear conditioning (FC) extinction training in mice Western blot	BDNF↑, mTOR↑	Psilocybin reversed FC-associated BDNF and mTOR reduction 7 days after administration.
Davoudian et al., 2023 doi:10.1021/acschemneuro.2c00637 [61]	Mice received either saline (10 mL/kg), ketamine (10 mg/kg) or psilocybin (1 mg/kg). c-Fos^GFP^ transgenic mice Whole-brain serial two-photon microscopy Light sheet microscopy	c-Fos↑ in anterior cingulate cortex, locus coeruleus, primary visual cortex, central amygdala, basolateral amygdala, hippocampus, medial habenula, lateral habenula, claustrum and anterior and midline thalamic nuclei, after both psilocybin and ketamine. c-Fos↑ in reticular nucleus of the thalamus, caudoputamen, periaqueductal gray and c-Fos↓ in dorsal raphe and insular cortex after psilocybin.	Psilocybin acutely modulates expression of c-Fos across different areas of the brain similarly to ketamine, but with some difference in dorsal raphe, insular cortex and hippocampus.
Rijsketic et al., 2023 doi:10.1038/s41386-023-01613-4 [62]	Mice were administered saline or psilocybin (2 mg/kg, i.p.), before placement into their home cage or an enriched environment. After 2 weeks, mice were administered saline or psilocybin and confined to either environment for 2 h. Light sheet fluorescence microscopy	c-Fos↑ in subregions of the neocortex, caudoputamen tail, central amygdala. c-Fos↓ in the hypothalamus, cortical amygdala, striatum and pallidum.	Both psilocybin and environmental context acutely modulate expression of c-Fos across different areas of the brain, in a mainly additive manner.
Funk et al., 2024 [64] doi:10.1016/j.neuroscience.2024.01.001	Male rats were randomized into four groups (vehicle or subcutaneous psilocybin 0.1, 0.5, 3 mg/kg at 1 mL/kg). Immunohistochemistry	c-Fos↑ in neurons and oligodendrocytes in the frontal cortex, nucleus accumbens, central amygdala, basolateral amygdala, locus coeruleus.	Psilocybin acutely increases the expression of c-Fos both in neurons and oligodendrocytes across several brain areas.
Shahar et al., 2024 [65] doi:10.1038/s41380-024-02477-w	Adult male mice were randomized into three groups (vehicle, psilocybin 4.4 mg/kg intraperitoneally or psychedelic mushroom extract (PME) containing the same amount of psilocybin). Western blot	Hippocampus: GAP43↑, synaptophysin↑, SV2A↑ (only in PME group). Amygdala: synaptophysin↑, SV2A↑ (only in psilocybin group). Frontal cortex: GAP43↑ Striatum: synaptophysin↑ (only in PME group).	Psilocybin and psychedelic mushroom extract both induce gene expression associated with neuroplasticity 11 days after administration.
Lerer et al., 2024 [66] doi:10.3389/fphar.2024.1391412	Male mice were randomized into three groups (vehicle, psilocybin 4.4 mg/kg or PME with equal dose of psilocybin). Real-time qPCR	c-Fos↑, egr1↑ in somatosensory cortex.	Both psilocybin and psychedelic mushroom extract acutely increase immediate early genes expression in somato-sensory cortex.
Zhao et al., 2024 [68] doi:10.1177/02698811241249436	Male mice, previously exposed to glucocorticoids for 21 days (CORT-induced depression model), were randomly divided into four groups (vehicle, 0.1 mg/kg, 1.0 mg/kg and 2.5 mg/kg psilocybin intraperitoneal injection). The same randomization was conducted on a population not exposed to glucocorticoids. Western blot Immunofluorescence	BDNF↑, TrkB↑, mTOR↑, Doublecortin↑ in the prefrontal cortex and the hippocampus.	Psilocybin reversed the reduction in neuro-plasticity biomarkers induced by chronic glucocorticoid exposure.

#### 3.1.2. DMT-Related Compounds

A total of 13 preclinical papers examining the psychoplastogenic effects of DMT, its substitutes and ayahuasca (a mixture of naturally derived harmaline alkaloids and DMT-related compounds), were included, all of which were published from 2017 onwards. A proteomic analysis, using high-definition mass spectrometry in human cerebral organoids treated with 5-MeO-DMT, revealed a significant upregulation of proteins involved in synaptic plasticity (e.g., NMDAR, CaMK2, CREB) and plasticity-related cytoskeletal reorganization (e.g., EFNB2, EPHB, CDC42), together with an inhibition of the pro-inflammatory NF-κB signaling pathway [73]. A single systemic or intra-ventral tegmental area (VTA) administration of 4-AcO-DMT could switch the motivational state of rats from opiate-dependent to a naïve-like non-dependent state, even after chronic opiate exposure or intra-VTA administration of BDNF, also reducing the aversive salience of withdrawal [74]. A further study, encompassing a combination of in vitro neuronal cultures, in vivo animal models and behavioral assessments, demonstrated that DMT, similarly to LSD and DOI, enhances structural (spinogenesis, synaptogenesis) and functional plasticity (increased frequency and amplitude of spontaneous excitatory postsynaptic currents) through intracellular 5-HT2A receptor activation [39].

Another experiment showed that a single intracerebroventricular injection of 5-MeO-DMT induced cell proliferation and increased dendritic complexity in the granule cells of the adult mouse dentate gyrus [75]. Chronic, intermittent low doses of DMT over two months was shown to facilitate fear extinction learning in Sprague Dawley rats, again suggesting a reopening of plasticity, with no influence on working/short-term memory or social interactions [76]. A controlled in vivo study on middle cerebral artery occlusion (MCAO) in rats showed that DMT significantly reduced ischemic brain lesion volume, improved motor function recovery, increased BDNF levels and decreased pro-inflammatory cytokines [77]. Ayahuasca was demonstrated to attenuate anxiety-like behavior during ethanol withdrawal in mice, possibly by preventing ethanol-induced increase in 5-HT1a receptor and prodynorphin levels in the hippocampus [78]. In another study, ayahuasca weakened fear memory reconsolidation in Wistar rats, with effects lasting up to 22 days and preventing fear reinstatement [79].

Rats exposed to predator stress and treated with DMT and pharmahuasca (the pharmaceutical version of ayahuasca) exhibited an upregulation of genes associated with synaptogenesis in the prefrontal cortex and hippocampus, along with a significant reduction of NF-κB2 expression [80]. The use of in vitro cultures of neuronal cells demonstrated that the structural neuroplastic effects of DMT (essentially in terms of dendritogenesis and spinogenesis) are largely mediated by intracellular 5-HT2A receptors, eventually prompting that DMT itself could be the endogenous ligand of this subpopulation of receptors [81]. An in vivo imaging study using two-photon microscopy showed that 5-MeO-DMT intensified dendritic spine formation in the mouse medial frontal cortex, resulting in a long-lasting increase in spine density [82]. A study using a combination of pharmacological (ketanserin) and genetic tools (5-HT2AR knockout mice) in vivo and in vitro provided evidence that 5-HT2AR activation is necessary for the neuroplastic and antidepressant-like behavioral effect of 5-MeO-DMT [83]. Finally, ayahuasca was found to significantly facilitate conditioned fear extinction in rats through a mechanism dependent on the activation of 5-HT2A and 5-HT1A receptors in the infralimbic cortex [84].

**Table 2:** Gene expression studies: Ayahuasca, DMT and related compounds.

References	Methods	Results	Comments
Dakic et al., 2017 [73] doi:10.1038/s41598-017-12779-5	Human embryonic stem cells were induced towards neural differentiation, expanded and grown into cerebral organoids; organoids were separated into a group treated with 5-methoxy-N,N-dimethyltryptamine (5-MeO-DMT) and a vehicle group. qPCR Immunohistochemistry	360 downregulated and 574 upregulated proteins, predicting dendritic spine and cellular protrusion formation, microtubule and cytoskeletal organization, mild T lymphocyte differentiation and inhibiting neurodegeneration, cell death and brain lesion *NMDAR*↑, *CaMK2*↑, *CREB*↑, *ERK1/2*↑ *NF-κB*↓, *mGluR5*↓, *PKC*↓, *PLC*↓, *CaM*↓, *AC1/8*↓, *IP3R*↓, *EPAC1*↓, *PKA*↓	5-MeO-DMT caused molecular alterations in human cerebral organoids associated with neuroplasticity and anti-inflammatory mechanisms.
Ly et al., 2018 [39] doi:10.1016/j.celrep.2018.05.022	Cortical neurons were randomized into ANA-12-treated group, rapamycin, ketanserin, control, then each group was randomized into 2,5-Dimethoxy-4-iodoamphetamine, N,N-Dimethyltryptamine or LSD for 24 h. Droplet digital PCR. ELISA.	Psychedelics caused a non-significant increase in BDNF expression, which was not seen in neurons pre-treated with ANA-12. Treatment with rapamycin or ketanserin blocked psychedelic-induced neuritogenesis.	Inhibiting TrkB, mTOR, 5-HT2A receptor blocked psychedelic-induced increase in neuritogenesis or BDNF expression, suggesting a role of these macro-molecular structures in the effects of psychedelics.
Nardai et al., 2020 [77] doi:10.1016/j.expneurol.2020.113245	Transient middle cerebral artery occlusion (MCAO) was induced in male rats before being randomized into 2 groups: i.p. bolus of vehicle or 1 mg/kg DMT followed by 2 mg/Kg in 24 h. qPCR. ELISA.	*APAF1*↑, *BNDF*↑, *IL-10*↑, *TNF-α*↓, *IL1-β*↓, *IL-6*↓	DMT reduced ischemic brain lesion volume and had a neurotrophic and anti-inflammatory effect, mediated by its modulation of cytokines and other neurotrophins.
Almeida et al., 2022 [78] doi:10.1016/j.bbr.2021.113546	Mice received 2.2 g/kg ethanol or saline i.p. injections every other day for nine days and then randomized into two groups (daily administration of a dose of ayahuasca corresponding to 1.76 mg/kg of N,N-dimethyltryptamine, DMT, or water). Western blot	Ayahuasca dampened the increase of 5-HT1A receptor levels in the hippocampus after induction by ethanol. Ethanol decreased the dynorphin/prodynorphin ratio in the striatum, and treatment with ayahuasca partially reverted this reduction. Ayahuasca prevented the increase of prodynorphin in the hippocampus induced by ethanol.	Ayahuasca attenuated anxiety-like behaviour, proposedly preventing ethanol-induced increases in 5-HT1A receptor and prodynorphin levels in the hippocampus.
Kelley et al., 2022 [80] doi:10.1021/acschemneuro.1c00660	A population of male rats was randomized into placebo or a 30-day stress regiment followed by administration of DMT 2 mg/kg i.p., harmaline 1.5 mg/kg i.p., DMT 2 mg/kg and harmaline 1.5 mg/kg i.p. combined (pharmahuasca), or vehicle every other day for 5 days. Electron paramagnetic resonance spectroscopy Real-time PCR	**DMT + Harmaline** *mitochondrial transcripts*↓, *CYBA*↓, *CYBB*↓, *p47phox*↓, *Rap1*↓, *Il1r1*↓, *Il1α*↓, *Tlr4*↓, *Tlr6*↓, *Tlr7*↓, *Ifngr1*↓, *NF-κβ2*↓, *Nrf2*↓, *Keap1*↑, *IPA Oxidative Phosphorylation signaling pathway*↓ **DMT** *Mitochondrial transcripts*↓, *Rap1*↓, *Il1r1*↓, *Il1α*↓, *Tlr4*↓, *Tlr6*↓, *Tlr7*↓, *Ifngr1*↓, *Lrp8*↑, *Ntrk2*↑, *Ntrk3*↑, *mTORC1*↑, *mTORC2*↑, *Crebbp*↑, *Nrf2*↓, *Keap1*↑ **Harmaline** *Mitochondrial transcripts*↓↑, *Rap1*↓, *CYBB*↓, *Il1r1*↓, *Il1α*↓, *Tlr4*↓, *Tlr6*↓, *Tlr7*↓, *Ifngr1*↓, *IPA synaptogenesis pathway*↑, *Sst*↑, *Sstr1*↑, *Sstr2*↑, *Sstr3*↑, *Sstr4*↑, *Nrf2*↓, *Keap1*↑, *IPA Oxidative Phosphorylation signaling pathway*↓	All treatments reversed some differentially expressed genes observed in PTSD, each with differences. All treatments reduced expression of ROS production-associated pathways, increased expression of anti-inflammatory and synaptogenesis-associated pathways.

#### 3.1.3. LSD

The analysis of the literature permitted us to identify 12 preclinical articles on the psychoplastogenic effects of LSD administration in animal models. The oldest one was focused on the influence of psychedelics on gene expression in rat brains. Using DNA microarrays and RNase protection assays on the prefrontal cortex, hippocampus and midbrain, the authors noted a significantly increased expression of seven genes involved in the mechanisms of synaptic plasticity, glutamatergic signaling and cytoskeletal architecture (*c-fos*, *arc*, *krox-20*, *NOR1*, *Ikβ-α*, *sgk*, *ania3*) after the intraperitoneal administration of 1 mg/kg of LSD, compared to control animals [85]. The next year, the same authors, with the aid of specific 5-HT1A and 5-HT2A antagonists (WAY100635 and MDL100907, respectively), not only confirmed their previous findings, but also first demonstrated that LSD-induced gene expression is largely a 5-HT2A-dependent phenomenon [86]. In 2004, with the use of microarray screening, RNase protection and real-time (RT)-PCR, they identified three new LSD-induced genes: *CCAAT enhancer binding protein β* (*C/EBP β*), *map kinase phosphatase-1* (*MKP-1*) and *induced by lysergic acid diethylamide-1* (ILAD-1). Again, the WAY100635 did not alter the expression trend of these genes, while MDL100907 partially hindered the effects of LSD on the expression of these genes [87].

An immunohistochemistry study with anti-BrdU (5-bromo-2-deoxyuridine) antibodies found no effect of LSD, following either acute or chronic administrations, on the fraction of proliferating cells in the adult rat hippocampus, while the sustained administration of a 5-HT2A antagonist (ketanserin) significantly augmented the proliferation rate [88]. An RNA-sequencing and qPCR experiment conducted on a sample of rats administered LSD (0.16 mg/kg) for 90 consecutive days, as compared to saline-injected controls, detected a significant difference in the expression of genes involved in synaptic plasticity (*Nr2a*, *Krox20*), neurotransmission (*Drd2*, *Gabrb1*) and neuropeptide signaling (*Npy*, *Bdnf*), [89]. A more recent study, already cited for DMT, used structured illumination microscopy (SIM) to test the psychoplastogenic effects of several serotonergic psychedelics. The treatment of rat neuronal cell cultures showed that LSD had the greatest capacity to promote neuritogenesis, spinogenesis and synaptogenesis. Once more, the use of selective inhibitors of 5-HT2A receptors lessened the increase in plasticity-related parameters induced by classic psychedelics [39]. The same research group with the same methodology demonstrated that transient exposures (from 15 min to 6 h) is sufficient to induce neuroplastic changes (neuritogenesis, spinogenesis and synaptogenesis) comparable to that observed following more prolonged periods of exposure (72 h) applied in the previous experiment. These changes persist after LSD has been removed from the organism and require a stimulation phase, mediated by the activation of TrkB receptor, and a growth phase, involving the sustained activation of mTOR and the AMPA receptor [90]. A more recent study investigating the co-expression networks in the prefrontal cortex via RNA-sequencing in rats, treated with daily administrations of LSD 0.16 mg/kg intraperitoneally for 90 days, reported an increased transcriptional entropy that resembled the gene expression profile of stem cells, likely reflecting a highly neuroplastic state [91]. The analysis of the differential expression of proteins using liquid chromatography–mass spectrometry of brain organoids originating from pluripotent human cells (iPSCs) and treated for 24 h with 10 nM of LSD (3.23 ng/mL) or vehicle, highlighted a significantly increased expression of a pool of proteins involved in neural remodeling (axon guidance, synaptic vesicle cycling, long-term potentiation) while providing evidence for the central role of mTOR in the determination of LSD-induced neuroplastic processes [92].

A whole-genome bisulfite sequencing (WGBS) and proteomic profiling study sampling rat prefrontal cortex found that the repeated administration of LSD (30 μg/kg/day) during 7 days significantly modulated the methylation of 635 CpG sites and the expression levels of 178 proteins involved in signaling pathways for the development of the nervous system, axon guidance, synaptic plasticity, quantity and cell viability of neurons and protein translation [93]. Recently Moliner et al. demonstrated, using a combination of several experimental techniques, that LSD exerts some neuroplastic effects independently of 5-HT2A-mediated signaling, directly binding to TrkB receptors and functioning as positive allosteric modulators of BDNF-TrkB signaling [94]. As already mentioned above, a study using an sCPP model in mice showed that a single intraperitoneal injection of LSD at a dosage of 1 μg/kg induced a critical period reopening, whose duration (about three weeks) was longer than that of ketamine (about 48 h), MDMA and psilocybin (about two weeks), being second only to ibogaine (longer than four weeks) [63].

**Table 3:** Gene expression studies: LSD.

References	Methods	Results	Comments
Nichols & Sanders-Bush, 2002 [85] doi:10.1016/S0893-133X(01)00405-5	Rats were randomized into 1 mg/kg of LSD i.p. or control. DNA microarrays RNase protection assays	Prefrontal cortex: c-fos↑, arc↑, krox-20↑, NOR1↑, Ikβ-α↑, sgk↑, ania3↑ Midbrain: c-fos↑, NOR1↑, Ikβ-α↑, sgk↑, ania3↑ Hippocampus: c-fos↑, krox-20↑, Ikβ-α↑, sgk↑	LSD increased expression of seven genes involved in synaptic plasticity, glutamatergic signaling and cytoskeletal architecture, in a region-specific manner.
Nichols et al., 2003 [86] doi:10.1016/s0169-328x(03)00029-9	Main experiment: rats were randomized into 1 mg/kg of LSD i.p. or saline i.p. Secondary experiments: rats were randomized into WAY100635 1 mg/kg+ LSD 1 mg/kg i.p, or WAY100635 1 mg/kg i.p alone, MDL100907 1 mg/kg + LSD 1 mg/kg i.p, or MDL100907 1 mg/kg i.p alone. DNA microarrays RNase protection assays and qPCR	c-fos↑, arc↑, krox-20↑, NOR1↑, Ikβ-α↑, sgk↑, ania3↑, homer1a↑ WAY100635 did not inhibit LSD-induced gene expression. MDL100907 inhibited LSD-induced gene expression.	LSD-induced expression of neurotrophism-associated genes in prefrontal cortex, and it appears to be largely mediated by 5-HT2A receptors.
Nichols & Sanders-Bush, 2004 [87] doi:10.1111/j.1471-4159.2004.02515.x	Rats were randomized into 1 mg/kg of LSD i.p. or saline i.p. In another experiment rats were randomized into WAY100635 1 mg/kg+ LSD 1 mg/kg i.p, or WAY100635 1 mg/kg i.p alone, MDL100907 1 mg/kg + LSD 1 mg/kg i.p, or MDL100907 1 mg/kg i.p alone. DNA microarrays RNase protection assays and qPCR	Prefrontal cortex: C/EBP β↑, MKP-1↑, ILAD-1↑ WAY100635 did not inhibit LSD-induced gene expression. MDL100907 inhibited LSD-induced gene expression. Hippocampus: MKP-1↑, ILAD-1↑ Midbrain: C/EBP β↑, MKP-1↑	LSD-induced expression of neurotrophism-associated genes in prefrontal cortex, and it appears to be largely mediated by 5-HT2A receptors.
Martin et al., 2014 [89] doi:10.1016/j.neuropharm.2014.03.013	Rats were randomized into saline or 0.16 mg/kg LSD i.p., every other day, for 90 days. qPCR	28 days after the final treatment, 283 transcripts exhibited differential expression (>25%) (*p* < 0.05), including: Gabrb1↑, Gabrb2↑, Gabrg3↑, Slc6a13↓, NR2a↑, NR2b↑, Bdnf↑, Krox20↑, Drd1↓, Drd2↓, Ndufs8↓, Ndufb2↓, Ndufb7↓, Nudufa1↓, Atp6v0b↓, Atp6v0e2↓, Atp6v0c↓, Atp5d↓, Cox7a2↓, Cox8a↓, Cox4nb↓, Gstt2↓, Gstp2↓	Chronic LSD administration causes long term changes in expression of genes implicated with a large variety of functions, including synaptic transmission, synaptic plasticity, cell projection organization, cell-cell signaling, cytoskeleton.
Ly et al., 2018 [39] doi:10.1016/j.celrep.2018.05.022	Cortical neurons were randomized into ANA-12-treated group, rapamycin, ketanserin, control, then each group was randomized into 2,5-Dimethoxy-4-iodoamphetamine, N,N-Dimethyltryptamine or LSD for 24 h. Droplet digital PCR ELISA	Psychedelics caused a non-significant increase in BDNF expression, which was not seen in neurons pre-treated with ANA-12 Treatment with rapamycin or ketanserin blocked psychedelic-induced neuritogenesis	Inhibiting TrkB, mTOR, 5-HT2A receptor blocked psychedelic-induced increase in neuritogenesis or BDNF expression, suggesting a role of these macro-molecular structures in the effects of psychedelics.
Savino & Nichols, 2022 [91] doi:10.1111/jnc.15534	Rats were randomized into saline or 0.16 mg/kg LSD i.p., every other day, for 90 days. RNA sequencing	Enriched gene ontology (GO) categories: “dendrite development”, “circadian rhythms”, “covalent chromatin modification/histone modification”. Weighted gene co-expression network analysis (WGCNA): six clusters showed differential activity between LSD and controls: GTP binding↓, serine hydrolase↓, ribosome↓, chromatin organization↑, vesicle mediated transport in synapse↑, cell-cell adhesion↑. Increased signaling entropy and reduced between sample entropy	LSD induced differential expression in genes involved in neural function and remodelling. LSD increased plasticity-like transcriptional entropy (signaling entropy) and reduced ageing-like transcriptional entropy (between sample entropy).
Ornelas et al., 2022 [92] doi:10.1016/j.expneurol.2022.114148	Brain organoids originating from induced pluripotent human stem cells (iPSCs) were randomized into LSD 10 nM (3.23 ng/mL) in 24 h or vehicle. Liquid chromatography–mass spectrometry (LC-MS) based proteomics	Out of 3448 identified proteins, 234 had significant LSD-induced modifications in expression (*p* < 0.05). Enrichment analysis for predicted affected pathways and biological processes (Metascape) found several processes potentially affected: DNA replication, axon guidance, synaptic vesicle cycle, mTOR pathway, long-term depression (LTD) and dopamine neurotransmitter release cycle.	LSD induced differential expression in genes involved in neuroplasticity. In particular, proteins involved in mTOR signaling pathway was significantly (*p* = 0.034) over-represented in the LSD-treated set (2.15%) compared to the control set; (0.79%).
Inserra et al., 2022 [93] doi:10.1016/j.pnpbp.2022.110594	Male rats were randomized into LSD 30 µg/kg/day for 7 days or vehicle. Whole-genome bisulfite sequencing Proteomic profiling	Modulation of 635 CpG site methylation, especially in autosomes Modulation of expression of 178 proteins involved in signaling pathways for the development of the nervous system, axon guidance, synaptic plasticity, quantity and cell viability of neurons and protein translation.	LSD modulated methylation of 635 CpG sites and induced differential expression in genes involved in neuroplasticity.

#### 3.1.4. Mescaline and Related Compounds

Only one study involving mescaline and other alkaloid compounds (e.g., N-methylmescaline, N,N-dimethylmescaline or trichocereine) derived from the giant columnar cactus *Trichocereus terscheckii* was considered relevant for our review [95]. This study evaluated the effects of these alkaloids on gene expression, using a cactophilic fly (*Drosophila buzzatii*) as an animal model. The authors examined the differential genomic expression of *Drosophila buzzatii* raised on *T. terscheckii* compared to its usual host (*Opuntia sulphurea*), as well as under higher doses of alkaloids. Under the alkaloid-enriched condition, upregulated genes included those involved in crucial neuronal processes such as neurotransmitter clearance, serotonergic mechanisms and general metabolism. The authors used a list of differentially expressed genes in flies to search for orthologous sequences in the human genome. They compared gene expression in native South American populations traditionally consuming hallucinogenic cacti (Aymara and Quechua) with control populations (Yukpa, Bari, Wichi and Yanesha). They revealed an over-expression of certain genes involved in neuroplasticity processes such as neurotransmitter regulation (e.g., *ATF4*, *ASIC1*) and nervous system development (e.g., *ATF4*, *ATP2A1*, *PARD3*, *LSAMP*, *DSCAM*, *TENM3*, *EEF2*, *CTSV*, *CTSF*). Conversely, some other genes linked to neurotransmitter regulation, including catecholamines (e.g., *ACTB*, *PEBP1*, *DBI*), nervous system development (e.g., *APOB*, *ATP2A1*, *DSCAM*, *RIDA*, *SPINT2*) appeared downregulated.

#### 3.1.5. Synthetic 5-HT2A Agonists (Such as DOI and 25C-NBOMe)

The earliest study included in this review demonstrated that DOI significantly augmented the long-term potentiation induced by tetanic stimulation (20 Hz for 20 s) of isolated rat sympathetic ganglia [50]. Conversely, an in vitro study in rat visual cortex slices showed that DOI inhibited tetanus-induced long-term potentiation in a concentration-dependent fashion through a mechanism seemingly dependent on 5-HT2A receptors and phospholipase C [51]. DOI was also able to significantly increase the transcription of *Arc*, encoding for a cytoskeleton-associated protein known to be contributory to structural neuroplasticity, as shown by immunohistochemistry [49] and supported by the findings of another study providing evidence for a BDNF-dependent mechanism of *Arc* induction [94]. The same technique, using anti-BrdU (5-bromo-2-deoxyuridine) antibodies, found no effect of DOI on the number of proliferating cells in the adult rat hippocampus, while sustained ketanserin administration was effective in increasing the proliferation rate [88]. Another in situ hybridization histochemistry study confirmed the results of Pei et al. [49], providing evidence for a BDNF-dependent mechanism of *Arc* induction [96]. An in vitro patch-clamp study showed that DOI reduced the spontaneous firing rate of cultured cortical neurons, increasing their input resistance [97].

One of the largest and most cited inquiries on the subject from the group of David E. Olson [39] soundly demonstrated that DOI, similarly to LSD and DMT, is able to induce structural plasticity (neuritogenesis, spinogenesis and synaptogenesis) through a mechanism dependent on 5-HT2A receptors, TrkB receptors and mTOR complex signaling. Consistently, DOI was later demonstrated to induce a long-lasting depression of excitatory currents mediated by quisqualate receptors (α-amino-3-hydroxy-5-methyl-4-isoxazolepropionic acid receptors or AMPA) in the prefrontal cortex of rats, presenting evidence in support of a 5-HT2A-dependent mechanism [98]. A multimethod investigation reported that a single DOI administration in mice could speed up fear extinction, raise cortical dendritic density and modify the epigenomic regulation of several enhancer regions involved in the expression of synaptogenesis-related genes [99]. Another multimethod experiment using WB, qPCR, in situ hybridization and immunofluorescence demonstrated an increased expression of *Arc*, *Bdnf1*, *Cebpb*, *cFos*, *Egr1* and *Egr2* and a significant rise in the levels of activated (phosphorylated) cAMP response element (CRE) binding protein (CREB), both dependent on MAPK (mitogen-activated protein kinase) and CaMKII (Ca^2+^/calmodulin-dependent protein kinase II) signaling [100].

A recent ex vivo whole-cell recording experiment noted that 25C-NBOMe significantly increased both spontaneous and evoked activity in cortical pyramidal neurons, globally facilitating glutamatergic transmission [101]. A single short report showed that DOI is capable of influencing myelin plasticity, increasing myelin density in rat hippocampus 24 h after administration [102]. The recording of neuronal activity in the mouse medial prefrontal cortex highlighted that DOI influenced the activity-dependent levels of neural synchronization, promoting desynchronization and high frequency rhythms (gamma frequencies) not only in active behavioral states, but also in resting states [103]. In mice, DOI was shown to induce an enduring improvement in cognitive flexibility through a two-step reversal learning task, at least partly by increasing mice’s sensitivity to reward omissions [104]. For the sake of completeness, we underline that the results of references [70,71] were already summarized in the psilocybin paragraph.

**Table 4:** Gene expression studies: DOI and 25C-NBOMe.

References	Methods	Results	Comments
Pei et al., 2000 [49] doi:10.1016/s0028-3908(99)00148-3	Rats were randomized into DOI 0.2 mg/kg i.p., DOI 1 mg/kg, DOI 2 mg/kg, ketanserin 2 mg/kg + DOI 1 mg/kg. In situ hybridization histochemistry.	DOI: dose-dependent *Arc*↑ in orbital cortex, cingulate cortex, frontal cortex, parietal cortex, less significantly in striatum; ketanserin completely inhibited DOI’s effects.	DOI increased Arc expression in many brain areas.
Benekareddy et al., 2013 [96] doi:10.1017/S1461145712000168	Rats were randomized into DOI 8 mg/kg or saline i.p. injection. Induced BDNF KO mice and wild type mice were each randomized into immobilization stress or being left undisturbed. BDNF KO mice and wild type mice were each randomized into saline or DOI injection. In situ hybridization Immunohistochemistry	Immobilization stress and *DOI*: *Arc*↑ in the neocortex but not in the hippocampus *BDNF: Arc*↑ in the neocortex and the hippocampus Induced BDNF KO mice: *Arc*↓ in the neocortex but not in the hippocampus; reduced *Arc*↑ with acute immobilization or *DOI.*	DOI and immobilization stress increase *Arc* expression, which appears to be regulated by *BDNF*.
de la Fuente Revenga et al., 2021 [99] doi:10.1016/j.celrep.2021.109836	Mice (n = 6 for each condition) were injected (i.p.) with vehicle or DOI. Frontal cortex samples were collected at 24 h, 48 h, or 7 days after administration. High-resolution, cell-type-specific and low-input ChIP-sequencing; RNA-sequencing	DOI significantly induced differential modifications in enhancer activation (H3K27ac) and gene expression in several Gene ontology (GO) categories. Some of these modifications were long-lasting (persisting after seven days).	DOI induces both transcriptomic and epigenomic modifications compatible with an increased plasticity state, the latter being largely persistent. The findings of this study are in agreement with a central role of 5-HT2A receptors in psychedelic-induced plasticity.
Desouza et al., 2021 [100] doi:10.3389/fnmol.2021.790213	5HT2A KO mice and WT mice were randomized into i.p. DOI 2 mg/kg or vehicle. Male rats, CREBαδ KO mice and WT mice were randomized into i.p. DOI 8 mg/kg or vehicle. Another group of rats was randomized into electroconvulsive seizure treatment or sham. Immunofluorescence Western blot qPCR Chromatin immunoprecipitation In situ hybridization	2 mg DOI in mice: Arc↑, Bdnf1↑, Cebpb↑, cFos↑, Egr1↑ and Egr2↑ in neocortex These effects were inhibited by MDL100,907 or U73122 administration. KN-62 inhibited the effect on cFos and U0126 inhibited the effect on Egr1; both inhibited the effect on Arc, Bdnf1, Cebpb, Egr2. DOI effect on Bdnf1 and cFos were inhibited in 5HT2A KO mice, while the effect on Arc, Egr1 and Egr2 was reduced. pCREB↑ with DOI, inhibited by KN-62 and U0126 8 mg DOI in rats:Arc↑, Atf3↑, Atf4↑, Bdnf1↑, Cebpb↑, Cebpd↑, Egr1↑, Egr2↑, Egr3↑, Egr4↑, cFos↑, JunB↑, Nfkbia↑. Arc, Bdnf1, Cebpb and cFos, but not Egr1 and Egr2 expression, was associated with pCREB enrichment. Electroconvulsive seizure treatment increased expression of Arc, Bdnf1, Cebpb, cFos and Egr2 similarly to DOI. CREBαδ KO mice had no significant difference in 5-HT2A or 5-HT2C expression after DOI administration compared to WT mice. DOI-dependent increase in Arc expression was reduced in CREBαδ KO mice.	Neurotrophic effects of DOI appear to be mediated by 5-HT2AR and Gq-coupled PLC pathway leading, through MAPK and CaMKII signaling to an increased phosphorylation of CREB in the cortex.

**Table 5:** Preclinical structural plasticity studies.

Study	Methods	Results	Comments
Jha et al., 2008 [88] doi:10.1016/j.neulet.2008.06.028	DOI (8 mg/kg) or LSD (0.5 mg/kg) was administered through intraperitoneal (i.p.) injection either once (acute) or once daily for 7 days (chronic). Immunohistochemistry study, using anti-BrdU (5-bromo-2-deoxyuridine) antibodies to assess the proliferation of adult hippocampal progenitors	No observed effect of DOI or LSD on the number of proliferating cells in the adult rat hippocampus	DOI and LSD do not seem to exert mitogenic effects on hippocampal progenitors.
Ly et al., 2018 [39] doi:10.1016/j.celrep.2018.05.022	For in vitro studies, cells were treated with LSD, DOI and DMT at a concentration of 10 μM, 10 μM and 90 μM, respectively. For the ex vivo experiment, rats were administered DMT i.p. at a dose of 10 mg/kg. For in vitro assays, structured illumination microscopy (SIM); for ex vivo assays Golgi–Cox staining. Tests with inhibitors of 5-HT2A receptors (ketanserin), TrkB receptors (ANA-12) and mTOR complex (rapamycin) were performed.	DOI, LSD and DMT induce structural plasticity (neuritogenesis, spinogenesis and synaptogenesis). Among the psychedelics tested, LSD appeared to be the one with the greatest psychoplastogenic effects. Ketanserin, ANA-12 and rapamycin abolished these effects.	DOI, LSD and DMT seem to augment neuronal remodeling, and this finding is confirmed by different methods in vitro and ex vivo (only for DMT). Psychoplastogenic effects are largely dependent on 5-HT2A receptors, TrkB receptors and mTOR complex signaling.
Lima da Cruz et al., 2018 [75] doi:10.3389/fnmol.2018.00312	Adult mouse single intracerebroventricular iniection of 1 μL 5-MeO-DMT solution BrdU Labeling, BrdU immunohistochemistry, clustering Analysis of BrdU+ cells, confocal microscopy	Cellular proliferation and increased dendritic complexity in granule cells of the dentate gyrus	DMT seems to exert a mitogenic effects on hippocampal progenitors.
de la Fuente Revenga et al., 2021 [99] doi:10.1016/j.celrep.2021.109836	Single administration of DOI or vehicle in 5-HT2AR^−/−^ mice as compared to 5-HT2AR^+/+^ controls. 3D automated method for quantitative structural spine analysis.	Increased density of transitional dendritic spines, but not mature mushrooms spines, in 5-HT2AR^+/+^, but not in 5-HT2AR^−/−^ mice. A significantly greater magnitude of LTP was evident in DOI-treated compared to vehicle-treated mice over the same time course.	5-HT2ARs seem to be necessary for the plasticity-boosting effects of DOI. These latter could partly explain the LTP facilitatory properties of this compound.
Shao et al., 2021 [53] doi:10.1016/j.neuron.2021.06.008	Mice treated with 1 mg/kg intraperitoneal psylocibin compared to placebo. Two-photon microscopy	Increased size and density of cortical dendritic spines that appear within the first 24 h and last at least for a month. These effects were not blocked by ketanserin administration.	Psychedelics directly induce neuronal structures remodeling and growth in mice, allegedly through a 5-HT2AR-independent mechanism.
Ly et al., 2021 [90] doi:10.1021/acsptsci.0c00065	Treatment of rat neuronal cell cultures with 10 μM LSD. Structured illumination microscopy (SIM).	Transient exposures (from 15 min to 6 h) is sufficient to induce neuroplastic changes (neuritogenesis, spinogenesis and synaptogenesis) comparable to that observed following more prolonged periods of exposure (72 h) applied in the previous experiment.	Neuroplastic changes would persist after short exposures to LSD and would require a stimulation phase, mediated by the activation of TrkB receptor and a growth phase, involving the sustained activation of mTOR and the AMPA receptor.
Ko et al., 2023 [102] doi:10.1016/j.biopsych.2023.02.388	Injection of 3 mg/kg of DOI in wake-behaving mice compared to a vehicle-injected group. Miniature microscope to study hippocampal cells from mice that expressed the calcium sensor under the control of the oligodendrocyte precursor cell (OPC) promoter.	DOI is capable of influencing myelin plasticity, increasing myelin density in rat hippocampus 24 h after administration.	Not only does DOI exert neuroplastic effects on neurons, but it seems also to induce similar effects on oligodendrocytes.
Vargas et al., 2023 [81] doi:10.1126/science.adf0435	10 μM of 5-methoxy-N,N-dimethyltryptamine (5-MeO) to wild-type (WT) and 5-HT2AR knock- out (KO) mice. Golgi–Cox staining	Neuroplastic structural effects are observed, essentially in terms of dendritogenesis, largely mediated by intracellular 5-HT2A receptors.	Intracellular 5-HT2A receptors seem to have a crucial role in the neuroplastic effects of tryptamines.
Jefferson et al., 2023 [82] doi:10.1038/s41386-023-01572-w	In vivo two-photon microscopy of mouse medial frontal cortex after i.p. 5-MeO-DMT.	Intensification of dendritic spine formation in the mouse medial frontal cortex, resulting in a long-lasting increase in spine density	Tryptamines are able to increase cortical dendritic spine density in vivo.
Moliner et al., 2023 [94] doi:10.1038/s41593-023-01316-5	Multiple in vitro and in vivo/ex vivo experiments comparing LSD, psilocin and vehicle. Various techniques were employed, including binding assays, microscale thermophoresis assay, nuclear magnetic resonance spectroscopy, split-luciferase complementation assay, fluorescence recovery after photobleaching, real time qPCR, single-molecule localization microscopy, ELISA, Western blotting	LSD and psilocin bound TrkB with high affinity (LSD Ki = 3.38 ± 1.39 nM; Psilocin Ki = 673 ± 3.05 nM), Y433F and TrkA.TM, but not S440A mutation, impaired LSD binding. LSD and PSI induced a fast and long-lasting dimerization of TrkB but not in Y433F heterodimers. Pretreatment with ketanserin or M100907 did not prevent this effect. LSD potentiated the effect of low doses of BDNF.	Contrary to what was previously observed, LSD and psilocin might exert at least part of their neuroplastic effects regardless of 5-HT2A-mediated signaling, through a direct TrkB-dependent mechanism. LSD and psilocin bind to the transmembrane domain of TrkB and act as positive allosteric modulators.
Du et al., 2023 [60] doi:10.1097/CM9.0000000000002647	A single dose of psilocybin (2.5 mg/kg, i.p.) in mice, 30 min before extinction training in a fear conditioning (FC) protocol. Golgi staining for the dendritic complexity and spine density	Reduced decrease of hippocampal dendritic complexity and spine density induced by FC.	Psilocybin might be able to dampen the anti-plastic effects of stress, facilitating FC extinction.
Zhao et al., 2024 [68] doi:10.1177/02698811241249436	Psilocybin (0.1, 0.5, 1.0, 2.5, 5.0 mg/kg) i.p. injection in mice. Western blot, immunofluorescence, dendrite analysis after Golgi staining	A single administration of psilocybin can reverse the detrimental effects of chronic glucocorticoid exposure on structural plasticity (density and number of dendritic spines and branches).	Psychedelics might reverse anti-plastic processes known to be associated with chronic stress and restore the adaptive capacity of neurons.

**Table 6:** Preclinical behavioral studies.

Study	Methods	Results	Comments
Cameron et al., 2019 [76] doi:10.1021/acschemneuro.8b00692	Male and female Sprague Dawley rats treated with DMT·fumarate (1 mg/kg) over two months. Behavioral assays included elevated plus maze, forced swim test and fear conditioning.	DMT improved mood-related behaviors and fear extinction, particularly in females. No significant changes in anxiety or cognition. DMT induced a reopening of plasticity without any influence on working/short-term memory or social interactions.	Chronic low-dose DMT shows promise for mood improvement without cognitive deficits.
de la Fuente Revenga et al., 2021 [99] doi:10.1016/j.celrep.2021.109836	Adult male mice treated with DOI (2 mg/kg) via intraperitoneal injection. Behavioral tests included forced swimming test (FST), dark–light test, novel object recognition and contextual fear extinction. Synaptic plasticity was analyzed using histological and electrophysiological techniques in 5-HT2AR^+/+^ and 5-HT2AR^−/−^ mice.	DOI significantly reduced immobility time in the FST, indicating antidepressant-like effects. No effects were observed on anxiety or cognitive performance. DOI accelerated fear extinction in 5-HT2AR^+/+^ mice but not in 5-HT2AR^−/−^ mice. Enhanced synaptic plasticity was noted in the frontal cortex, characterized by an increase in dendritic spines. Enhanced synaptic plasticity was noted in the frontal cortex, characterized by an increase in dendritic spines.	DOI exhibits potential as an antidepressant, particularly in reducing behavioral despair without affecting anxiety. Its effects on fear extinction and synaptic plasticity via 5-HT2A receptor activation support its therapeutic potential for anxiety disorders.
Nardou et al., 2023 [64] doi:10.1038/s41586-023-06204-3	Social conditioned place preference (sCPP) test in adult male mice treated with psilocybin (0.3 mg/kg), LSD (1 µg/kg), ketamine (3 mg/kg), ibogaine (40 mg/kg) and saline as a control.	Psychedelics enhanced social reward learning in mice. The effects of psilocybin and LSD lasted 2–3 weeks; ibogaine lasted for 4 weeks and ketamine lasted for 1 week. Cocaine had no effect. Psilocybin induced a reopening of the critical period, with a duration similar to MDMA (approximately two weeks), longer than ketamine (approximately 48 h), but shorter than LSD (approximately three weeks) and ibogaine (longer than four weeks).	This suggests that psychedelics may facilitate social reward learning, with potential therapeutic applications in autism, PTSD and depression.
Werle et al., 2024 [84] doi:10.1111/bph.16315	Contextual fear conditioning in Wistar rats treated with ayahuasca (0.3 mg/kg of DMT). Antagonists for 5-HT2A and 5-HT1A receptors were infused into the infralimbic cortex.	Ayahuasca enhanced fear extinction without affecting anxiety or exploratory behavior. It facilitated conditioned fear extinction in rats through a mechanism dependent on the activation of 5-HT2A and 5-HT1A receptors in the infralimbic cortex. Effects were blocked by 5-HT2A and 5-HT1A receptor antagonists.	Ayahuasca enhances fear extinction without affecting anxiety or exploratory behavior. Effects are blocked by 5-HT2A and 5-HT1A receptor antagonists.
Šabanović et al., 2024 [104] doi:10.1038/s41380-024-02439-2	Male C57BL/6J mice treated with DOI (2 mg/kg). Behavioral assays included a probabilistic reversal learning task. Ex vivo MRI was used to assess brain structural changes 24 h post-treatment.	DOI enhanced cognitive flexibility, with mice adapting faster to task changes and showing novel strategies in response to reward omissions. MRI revealed increased brain volume in sensory and association areas, indicating structural plasticity.	DOI’s enhancement of cognitive flexibility and ability to modify learning strategies underscores its potential utility in treating conditions characterized by rigid cognitive patterns, such as OCD or addiction.
Kelly et al., 2024 [67] doi:10.1038/s41386-023-01744-8	Fear conditioning and extinction training in C57BL/6J mice treated with the psilocin analogue 4-Hydroxy-NN-diisopropyltryptamine (4-OH-DiPT) (1 mg/kg or 3 mg/kg). Anxiety tests included open field, light–dark box, elevated plus maze and novelty-suppressed feeding tests.	4-OH-DiPT promoted fear extinction and spontaneous inhibitory post-synaptic currents (sIPSC) in the basolateral amygdala. Females exhibited significant reductions in freezing and avoidance behaviors. No significant effects were observed in male mice.	Highlights the sex-dependent effects of 4-OH-DiPT on fear extinction and anxiety responses.
Hinchcliffe et al., 2024 [69] doi:10.1126/scitranslmed.adi2403	Affective bias test (ABT) in Lister hooded rats treated with psilocybin and ketamine. Additional substances, such as scopolamine, were also tested.	Psilocybin and ketamine altered affective bias, while high doses of ketamine induced cognitive impairment with slower decision-making. Psilocybin also demonstrated “re-learning effects”, not only reducing negative biases similarly to ketamine but also uniquely increasing positive biases.	Demonstrates dose-dependent effects of psilocybin on emotional processing and cognitive function.
Sekssaoui et al., 2024 [70] doi:10.1038/s41386-024-01794-6	WT mice subjected to chronic despair conditioning protocol were randomized into DOI 1 mg/kg, psilocybin 1 mg/kg, lisuride 1 mg/kg or vehicle i.p. injection. 5-HT2A knock-out mice subjected to chronic despair conditioning protocol were randomized into psilocybin 1 mg/kg i.p., WAY-100635 0.5 mg/kg + psilocybin 1 mg/kg i.p., SCH23390 0.03 mg/kg, s.c. + psilocybin 1 mg/kg i.p., eticlopride + psilocybin 1 mg/kg i.p., DOI 0.05 mg/kg/day for 6 days or psilocybin 0.05 mg/kg/day for 6 days. Behavioral assays included novelty-suppressed feeding, sucrose preference and forced swim test.	Psilocybin, DOI and lisuride improved depressive-like behaviors in WT mice. DOI and lisuride failed to induce these effects in 5-HT2A receptor knockout mice. Psilocybin induced antidepressant effects in 5-HT2A knock-out mice and in mice pretreated with WAY-100635, SCH23390 or eticlopride.	Findings suggests that the antidepressant effects of psilocybin, oppositely to DOI and lisuride, might be partly independent of 5-HT2A receptor activation.
Takaba et al., 2024 [71] doi:10.1007/s00210-023-02778-x	Male C57BL/6J mice (7–9 weeks old) were randomized into i.p. administration of psilocin, DOI, TCB-2 and vehicle. FST, TST and novelty-suppressed feeding test (NSFT) were performed 24 h after administration. Each test was repeated every week for a month after administration.	Treatment with psilocin, DOI and TCB-2 significantly reduced immobility times in the FST and TST, compared to vehicle.Volinanserin, a 5-HT2A antagonist, abolished these effects. The effects induced by psilocin endured for at least three weeks. Psilocin also reduced the latency to feed in the NSFT. Pretreatment with volinanserin did not diminish this efect. In contrast, DOI and TCB did not show this property.	Results show that the antidepressant and anxiolytic properties of psychedelics might involve different mechanisms. Only psilocin exerted anxiolytic effects in the NSFT, and these were not affected by volinanserin, suggesting a 5-HT2A-independent mechanism. Moreover, only psilocin produced sustained antidepressant effects in the FST and TST for three weeks.

### 3.2. Clinical Evidence

The clinical studies were presented in chronological order across the different compounds, on the unique basis of the research methodology, including neuroimaging, electrophysiology, biohumoral markers, studies devoted to behavioral correlates of neuroplasticity, and, finally, case reports.

#### 3.2.1. Neuroimaging

An MRI study demonstrated a reduced cortical thickness of the posterior cingulate cortex (PCC, a key node of the default mode network, DMN) in regular users of ayahuasca compared to controls, which was in turn significantly associated with higher scores in the self-transcendence dimension and all of its three subdimensions (self-forgetfulness, transpersonal identification and spiritual acceptance), as measured through the temperament and character inventory—revised (TCI-R) [105]. An increase in brain entropy (operatively definable as the predictability inverse of a functional MRI time–series) was detected in several brain areas, from primary sensory to associative transmodal networks, after the intravenous administration of 75 μg of LSD in a group of nineteen healthy adults, which was in turn predictive of an increased trait openness after 2 weeks, as defined by the revised NEO personality inventory (NEO-PI-R) [106]. Twelve healthy volunteers presented a decreased amygdala response to emotional stimuli 1 week after the administration of 25 mg/70 kg of psilocybin that returned to baseline levels after 1 month [107]. A study using a high-field 7 Tesla MRI among chronic ayahuasca users, compared to controls, described an increase in morphometric similarity in midline, temporal and prefrontal structures (e.g., cingulate, entorhinal, orbitofrontal, anterior insular cortices), together with a decrease in precentral and postcentral cortices. This finding would indicate an increased cytoarchitectonic differentiation in sensorimotor areas and a reduced structural differentiation in transmodal areas [108]. Finally, a recent study based on longitudinally repeated functional MRI scans in healthy subjects demonstrated that psilocybin profoundly altered brain functional connectivity, namely inducing an intra-network desynchronization within the DMN and inter-network uncoupling between DMN and other DMN-connected areas, particularly the anterior hippocampus [109].

#### 3.2.2. Electrophysiology

A magnetoencephalography controlled trial using an auditory oddball paradigm found that subjects administered LSD, when compared to subjects treated with placebo, show a dampened mismatch negativity to deviant stimuli and an increased negativity after standard stimuli, suggestive of a partial disruption of top-down predictive processes, both in the sense of error detection and sensory adaptation [110]. A polysomnography placebo-controlled experiment reported that psilocybin did not alter the majority of sleep-related parameters (sleep latency, total sleep time, sleep efficiency and the number of sleep cycles), although its administration was associated with an increased REM sleep latency and a reduced delta power during the first sleep cycle [111]. A double-blind, placebo-controlled trial enrolling patients diagnosed with major depressive disorder found that psilocybin increased auditory evoked theta power two weeks after a single dose administration, and that such an increase in theta frequencies correlated with the reduction of depression scores, as measured with the clinician-administered GRID-HAMD-17 [112]. Another electroencephalography study failed to show differences in event-related visual potentials (both before and after photic tetanus) between placebo-treated subjects and subjects administered 10 μg sublingual LSD, either acutely or after 14 doses of the drug over 6 weeks [113].

#### 3.2.3. Biohumoral Markers

A recent study enrolled 24 recreational psychedelic users and investigated the influence of LSD on blood BDNF levels through a within-subject placebo-controlled design. Subjects were administered either 5, 10 or 20 μg of oral LSD or placebo. BDNF levels were measured 2, 4 and 6 h after the administration. Although all of the three dosages tended to increase blood BDNF levels after 6 h, only 5 and 20 μg were significantly different from placebo. However, a non-significant trend towards a dose–response relationship was observed [114].

#### 3.2.4. Behavioral Studies

A study used a randomized, double-blind, placebo-controlled, crossover design to investigate the effect of 50 μg of LSD on neurocognitive functions including episodic memory (Rey-Osterrieth complex figure, object-location memory task, Rey auditory–verbal learning test), fluency (verbal fluency task, design fluency task), cognitive flexibility (Wisconsin card sorting test), sustained and shifting attention (trail-making test), inhibitory control (Stroop task) and perceptual organization (block design test). About a day after assumption, subjects treated with LSD showed improved visuospatial memory and phonological verbal fluency parameters compared to placebo (the “afterglow”), while presenting an impaired cognitive flexibility-related performance (the “hangover”) [115]. Another within-subject, placebo-controlled trial explored the impact of LSD (75 μg in 10 mL saline, administered intravenously) on flexible learning (specifically, through probabilistic reversal learning, or PRL, design) in 19 healthy volunteers. The results showed that LSD increased reward-related learning significantly more than learning after punishment and facilitated exploratory behaviors, both in terms of reduced stimulus stickiness (i.e., the repetition of previous choices, despite changes in reinforcement patterns) and increased reinforcement sensitivity during the reversal phase (i.e., the ability to adapt to changing reinforcement patterns) [116].

#### 3.2.5. Case Reports

Three case reports were included in this review. The first report is about a 35-year-old male patient that developed intractable phantom pain after the amputation of his right leg. Attempts of treatment with opioids, pregabalin and medical cannabis failed to attain a satisfactory and durable remission of pain symptoms. He underwent a trial with a single assumption of psilocybin-containing dried mushrooms, with a significant, albeit transient response of the painful phenomenology. The authors then proposed a protocol combining dried mushrooms with mirror visual feedback, managing to induce a long-standing remission of the phantom limb pain. They interpreted the facilitatory effect of psilocybin on mirror visual feedback as the result of an increased neuroplastic activity, allowing for cross-modal cortical reorganization [117]. Another case series describes three subjects suffering from chronic pain: a 37-year-old male patient with a spinal cord injury after a rollover accident, a 69-year-old female patient suffering from complex regional pain syndrome and a 40-year-old female patient with a lumbar radiculopathy secondary to intervertebral disk degeneration. All of these cases benefited from psilocybin-containing mushrooms at low, sub-hallucinogenic doses, but the third one attained persisting remission of pain symptoms after each mushroom ingestion, which lasted from two to eight weeks and tended to be more sustained when combined with physical exercise [118]. The last report deals with three cases of impaired olfaction, one due to a non-COVID-19 (coronavirus disease 19) respiratory infection, a second due to COVID-19 respiratory infection and a third presenting idiopathic anosmia since childhood. The first two cases reported a persisting amelioration of their olfactory deficit after the assumption of psilocybin-containing mushrooms, while the third one attained a significant, although transient benefit after assuming 100 μg of LSD [119].

## 4. Discussion

Preclinical evidence seems consistent in demonstrating that psychedelics exert plasticity-related properties through different levels of biological complexity. Gene expression studies (Table 1, Table 2, Table 3 and Table 4) show that these compounds can induce the expression of IEGs (e.g., *c-Fos* and *egr1*), which are known to be involved in cell growth, differentiation and adaptation, not only in neurons, but in putatively every nucleated cytotype [120]. Furthermore, the evidence hereabove gathered indicates that psychedelics induce the expression of specific genes related to neuronal differentiation and functioning, including genes known to be involved in synaptic plasticity (*Nr2a*, *Krox20*), neurotransmission (*Drd2*, *Gabrb1*), neuropeptide signaling (*Npy*, *Bdnf*), cytoskeleton remodeling (*Arc*) and so forth.

The evidence coming from transcription and translation studies is in agreement with microscopy experiments (Table 5) replicatedly observing an increase in neuro-remodeling morphological markers (neuritogenesis, spinogenesis, synaptogenesis) after exposures to psychedelic compounds, in vitro, in vivo and ex vivo. The evidence concerning hippocampal neurogenesis seems to be more controversial, with some experiments reporting no influence of psychedelics on progenitor proliferation [88] and others recording a significant increase in proliferative rates [75]. To resolve these inconsistencies, some authors suggest that hippocampal neurogenesis might be a more typical 5-HT1A-mediated effect of chronic treatments with SSRIs, while psychedelics would be more associated with cortical plasticity [121,122]. Interestingly, neuroplastic effects might also benefit oligodendrocyte functioning and myelin turnover [102]. Moreover, these properties might not only be relevant to basal conditions, being also effective in reversing anti-neuroplastic conditions induced by experimental models of chronic stress (e.g., glucocorticoid exposure) [60,68].

Behavioral studies (Table 6) included learning assays (e.g., conditioned fear extinction, probabilistic reversal learning tasks, social conditioned place preference), classical models of depressive symptoms (forced swimming test or FST, tail-suspension test or TST) and anxiety (novelty-suppressed feeding test or NSFT), along with the affective bias test. All of these studies report findings in agreement with behavioral correlates of a neuroplastic effect of the compounds investigated. In particular, DMT, psilocybin and DOI were shown to be effective in reducing the timing for conditioned fear extinction. Both DOI and psilocybin were demonstrated to reduce immobility time in FST and TST models of depression. Interestingly a non-psychedelic 5-HT2A agonist, lisuride, was demonstrated to exert similar effects on depressive-like behaviors in murine models. By means of multiple strategies (e.g., 5-HT2A knock-out mice, 5-HT2A antagonist assays), these effects were shown to be strictly dependent on 5-HT2A-mediated neurotransmission for DOI, DMT, TCB-2 and lisuride, whereas psilocybin/psilocin exerted some antidepressant-like and anxiolytic-like (i.e., reduced feeding latency at the NSFT) effects also when 5-HT2A contribution was abolished.

Evidence from all of the three domains (genetic expression, microscopy imaging and behavioral assays) is consistent in demonstrating a central role of 5-HT2A receptors in psychedelic-induced neuroplasticity, perhaps with a peculiar contribution provided by the intracellular subpopulation of these receptors [81]. Indeed, most essays using pharmacological antagonists of 5-HT2A receptors, such as ketanserin, show a significant reduction of the neuroplasticity-related effects of psychedelics. Genetic engineering studies with 5-HT2A receptor knockout mice seem to confirm this finding in vivo and in vitro. However, psilocybin/psilocin and LSD might also induce antidepressant and psychoplastogenic effects bypassing 5-HT2A signaling, perhaps through a direct TrkB-mediated mechanism, as was recently described [94]. Furthermore, psychedelics, acting on 5-HT2A receptors located on pyramidal cells’ apical dendrites, increase glutamate-mediated neurotransmission through AMPA receptors [123].

Therefore, as already hinted at by previous accounts [124], psychedelic-induced BDNF-mediated hyperplasticity (PIBHYPE) might occur through three different mechanisms, which do not appear mutually exclusive: an “indirect pathway”, through an increased glutamatergic stimulation of AMPA/kainate receptors, similarly to ketamine; a “direct pathway” through the classical stimulation of 5-HT2A receptors; and (at present limited to psilocybin and LSD) a “hyperdirect pathway”, involving a direct stimulation of BDNF receptors (TrkB). As previously pointed out by Calvin Ly [90] and colleagues, these pathways might also be intended as a temporal consecution of neurobiological events, with a 5-HT2A/TrkB-dependent stimulation or induction phase (corresponding to the direct and hyperdirect pathways) and an AMPA/mTOR-dependent growth or modification phase (corresponding to the indirect pathway). The first phase would represent the acute metaplastic moment of psychedelic action, wherein the plastic potential of the brain is set to a primordial level similar to that of critical periods of plasticity. The second phase would instead underlie post-acute structural (e.g., neuritogenesis, spinogenesis, synaptogenesis) and functional (e.g., long-term potentiation and long-term depression) plasticity processes.

The most interesting finding deriving from the clinical literature hereabove summarized is the consistent finding of a decreased activation of the DMN and DMN-connected areas after exposure to psychedelics. Since the DMN is now deemed to be the neural substrate of self and self-reference representation (being, for instance, involved in internal activities like introspection, rumination and mind-wandering), its inhibition by psychedelics might correspond to the ego dissolution/self-transcendence/oceanic boundlessness experiences that constitute perhaps the more defining feature of psychedelic-induced phenomenology [125]. However, the most relevant findings to our topic are the reduced cortical thickness of PCC and the reduced cytoarchitectonic differentiation in transmodal areas among regular ayahuasca users [105,108]. These observations are at once intriguing, because they demonstrate in vivo in human subjects a macroscopic and persistent plastic change in brain structure and connectivity; and disquieting, given the importance of self-representational processes in psychology and psychopathology [126,127]. Weakening brain structures devoted to self/ego construction might be useful acutely, but seemingly disastrous chronically. Moreover, psychedelics might prove harmful in those conditions, which are typically characterized by baseline deficits in various complexity levels of self-representation (e.g., subjects at high risk for psychosis or subjects with a borderline personality organization) [121,128].

The results of the present review are in substantial accord with the REBUS (RElaxed Beliefs Under pSychedelics) model proposed by R.L. Carhart-Harris and K. J. Friston [129]. On the neurobiological level, the acute metaplastic phase (or stimulation phase according to Ly and Olson [90]) would correspond to the entropic state typical of psychedelic states and would allow the decanalization (relaxation) of previously established representations of self and the world (beliefs or generative models or priors); the post-acute morpho-functional plastic state would underlie the processes of restructuring or updating such aprioristic representations (recanalization) [130]. In the event that these had been dysfunctional or maladaptive, psychedelic treatment would thus enable the subject to break out of pathological canalizations and adopt new, hopefully more adaptive top-down predictive representations. The clinical cases included in this review might appear instructive in this regard, with psilocybin not only facilitating the recovery of olfaction in anosmic subjects [119] (i.e., a generic plastogenic effect), but aiding in the remission of the phantom limb pain, allegedly allowing for a plastic updating of bodily representational schemata (i.e., the restructuring of priors) [117].

Some significant open issues remain in psychedelic research, particularly in the domain of iPlasticity. First of all, the question whether the acute psychedelic phenomenology is necessary and/or sufficient for therapeutic effects. Some authors (“phenomenists”) maintain that, without these effects, no benefit occurs, being the psychedelic experience itself the core of therapeutic action [131]. Others (“non-phenomenists”) deem that a psychoplastogenic drug with the same neurobiological effects of psychedelics, but devoid of psychodysleptic properties, might equally attain the same clinical benefit [132]. In particular, Olson’s group [133] is investigating on iboga analogs (e.g., tabernanthalog or TBG) in murine models, with currently successful results, albeit lissencephalic animals might not be the best animal models for psychedelic therapy (in this regard, see [134]). Another possible interpretation we advance (“epifenomenist” view) is that the psychedelic experience might not be therapeutic per se, but the neurobiological processes responsible for beneficial outcomes could not be dissociable from acute psychodysleptic effects. Provided that neuroplasticity is currently emerging as the final common pathway of all antidepressant treatments [135], whether by a direct or an indirect stimulation of BDNF-TrkB signaling, a second question arises: could we circumvent the different and complex mechanisms of action of “antidepressants” and develop a direct TrkB agonist that works downstream of them-all? At the moment, the available evidence does not grant any operative answer [136].

Finally, there remains the problem regarding the transformative quality (salutogenic versus pathogenic) of neuroplasticity processes: while virtually every psychotropic substance generates plastic adaptive processes (post-acutely for psychoplastogens proper, after chronic exposure for most other psychotropics) [137], nothing precludes such changes from leading to more maladaptive system configurations, compared to the status quo ante (the “plastic paradox”, as is defined in [138]). For example, pathogenic plasticity processes are known to occur following repeated use of psychostimulants [139], along with after chronic exposure to antipsychotic drugs [140,141]. Plasticity processes are therefore “outcome-agnostic” and can take different diverging trajectories, some being dysfunctional tour court, others being functional in the short-term but dysfunctional in the long course, others more steadily world-adaptive [142]. The specific trajectory and its related clinical outcomes might depend on a multitude of variables pertaining to the set (e.g., trait and state psychopathological features), the setting (e.g., environmental context) and the drug (e.g., dosage, site and mechanism of action).

A limitation of this systematic review is that the collection and analysis of the clinical literature concerning psychedelic-induced neuroplasticity is not as exhaustive as the presentation of the pertaining preclinical literature. This is partly due to unavoidable methodological issues, since the clinical literature recovered with our research strategy is only inferentially related to the psychoplastogenic effects of psychedelics, whereas most of the preclinical experiences involved approaches convincingly suggesting a direct association between the psychoplastogenic effects and the modifications in the modelized behavioral surrogates of learning and affection. Nonetheless, we decided to include clinical literature as it provides an interpretive key for applying preclinical evidence to psychedelic-related psychopathology and neuropharmacology in humans.
